# Pandemic, power and paradox: Improvising as the New Normal during the COVID-19 crisis

**DOI:** 10.1177/13505076221132980

**Published:** 2023-02

**Authors:** Ace Volkmann Simpson, Alexia Panayiotou, Marco Berti, Miguel Pina e Cunha, Shireen Kanji, Stewart Clegg

**Affiliations:** Brunel University London, UK; University of Cyprus, Cyprus; University of Technology Sydney, Australia; Universidade Nova de Lisboa, Portugal; Brunel University London, UK; University of Sydney, Australia; University of Stavanger, Norway

**Keywords:** COVID-19, improvisation, paradox, power

## Abstract

The global COVID-19 pandemic made salient various paradoxical tensions, such as the trade-offs between individual freedom and collective safety, between short term and long-term consequences of adaptation to the new conditions, the power implications of sameness (COVID-19 was non-discriminatory in that all were affected in one way or another) and difference (yet not all were affected equally due to social differences), whereas most businesses became poorer under lockdown, others flourished; while significant numbers of workers were confined to home, some could not return home; some thrived while working from home as others were challenged by the erosion of barriers between their private and working lives. Rapid improvisational responding and learning at all levels of society presented itself as a naturally occurring research opportunity for improvisation scholars. This improvisation saw the arrival of a ‘New Normal’, eventually defined as ‘learning to live with COVID-19’. The five articles in this special issue capture critical aspects of improvisation, paradoxes and power made salient by the COVID-19 pandemic in contexts ranging from higher-education, to leadership, to medical care and virtue ethics. In their own ways, each breaks new ground by contributing novel insights into improvisation scholarship.

Improvisation, a recognized source of organizational learning and adaptation (Abrantes et al., 2022; Ciuchta et al., 2021; Cunha et al., 2017; Miner et al., 2001), has traditionally been viewed as an exception: organizations need to improvise *episodically*, when routines fail. Understood as relevant for organizations needing to increase capacity for responding to the unexpected (Weick, 1998), promoting organizational agility (Cunha et al., 2020; Cunha and Cunha, 2001) and responsiveness (O’Toole et al., 2021), reconceptualizing time (Crossan et al., 2005), cultivating resilience (Giustiniano et al., 2018, 2020) or learning-by-doing (Rerup, 2001), improvisation is normally represented as a localized practice: temporary, situated, responsive to some specific issue or threat bounded by time and space (Abrantes et al., 2020; Crossan et al., 2005). Then came the COVID-19 pandemic and the exception became the New Normal (although many non-Western countries had already experienced exceptions becoming the rule, through ongoing conflict (Afghanistan) epidemic (ebola in Sub-Saharan Africa), natural disaster (Tsunami and nuclear meltdown in Japan), etc.). Not only did the disruptions, caused by the rapid diffusion of the virus, trigger the need for quick adaptation (such as the need to work or study from home, or to guarantee income to people whose jobs could not be performed because of lockdowns) but also the improvised responses to these challenges produced additional complexity, often in the form of paradoxical tensions. For example, the Australian government’s adopting strict border controls for almost two years was instrumental in protecting the population from infection (to the point that the mortality rate in 2020–2021 was lower than usual), yet it also had the paradoxical effect of inducing large numbers of healthcare workers who had family overseas to leave the country, with the consequence that, as of 2022, the Australian health system is in a severe crisis caused by lack of medical staff. Beyond this specific example, the stress of working to protect and save lives from COVID-19 infection at the height of the pandemic has exacerbated a pre-existing shortage of staff in Western countries (Britnell, 2019).

## Insights into improvisation from the COVID-19 pandemic

The COVID-19 pandemic offered a supreme opportunity for revisiting and challenging organization-as-usual and exploring new ways of understanding management learning through improvisation. In line with the philosophy of *Management Learning* (critical, reflexive, imaginative, thought-provoking ideas), we sought to contribute to the understanding of improvisation in and around organizations by inviting authors to submit scholarship exploring themes of learning by fast responding, paradoxes of improvisation, leading to learning from the impact and implications for power relations of major disruptions, of learning from crises for relations of diversity and inequality as well as learning to respond with compassion and virtue, themes that have recently been receiving increasing scholarly attention (e.g. Tabesh and Vera, 2020).

The spread of the corona virus introduced rapid changes, giving rise to many types of improvisation, making the world a massive improvisational experiment. Improvised responses were seen across every sector but with different implications for the people affected within and between sectors. Healthcare saw the introduction of pop-up hospitals, new test kits, new vaccines and the repurposing of medicines and vaccines, working with or without personal protective equipment (PPE) in health and social care. Education incorporated new methods of online class delivery, collaboration and running exams that were online or, if in person, maintained social distancing. Supermarkets introduced special hours and deliveries for care workers and those who are elderly and vulnerable. Added risk was experienced by workforces, which faced relatively high exposure to the virus. Manufacturing saw the repurposing of production to deliver needed products such as protective equipment and ventilators. Distribution saw disruption to supply chains and working arrangements. Government introduced guarantees for a percentage of worker salaries (the United Kingdom, Denmark, Norway, New Zealand, Australia, etc.), along with a rise in absolutism and paternalism and a decline in democratic process seen both in national and corporate levels as individual freedoms were significantly restricted by leaders who took it on themselves to decide what others could and could-not do.

Interestingly, in face of the massive disruptions caused by the serial pandemics, improvisation gave rise to improvisation: the often-improvised policies imposed by governments caused an upheaval in the normal ‘rules of the game’, which induced businesses and not-for-profit organizations to improvise new arrangements and organizational forms, provoking, in turn, a number of improvised individual coping strategies. For example, imposition of social distancing rules forced companies to enable remote working, which led some individuals to reappraise their living arrangements, for instance, deciding to move away from cities. Such moves produced impacts on other organizations (e.g. additional pressures on rural areas service providers or severe decline in some businesses in the now half deserted central business districts), which required further improvisational strategies.

Improvised responses were also seen across aspects of the working environment. Working from home came to encompass larger sections of the workforce than had been the case prior to the COVID-19 pandemic, with many facing the challenges of working in shared home–work spaces with other household members, including children (creating a social, as well as a health crisis for women who still shoulder a disproportionate share of household work and childcare, although men’s share of childcare increased in many contexts; see Craig and Churchill, 2021). The impact of this experience in terms of the future organization of work and workspaces, including its gendered effects, is yet to be seen (e.g. Fayard et al., 2021). Team, group and community learning was expanded into virtual space through an increased use of online platforms facilitating improvisation in teams, communities of practice, pedagogy, grassroots innovation and social movements. The ways of responding to COVID-19 have undoubtedly lowered the priority of diversity initiatives in organizations, while individuals have been affected unequally within organizations, for example, by age, gender, race and ethnicity, disability, prior health status and occupation. White-collar jobs that relied on digital affordances were more likely to be retained through working from home arrangements while much essential work (in supermarkets, hospitals, care homes, transport, agriculture) was also retained and expanded but with greater exposure to risk of morbidity and mortality as dangerous work in terms of public health, further highlighting social inequalities. Compassion, in terms of ‘suffering together’, took on new meaning in notions of leadership and as individual-collective interdependencies.

The premise of this special issue is that the COVID-19 pandemic has provided an exceptional opportunity for understanding organizational learning through improvisation (Hadida et al., 2015), defined as purposeful action conducted in the absence of a plan using available resources (Cunha et al., 1999). The pandemic has produced innovations in improvisation. Typically, improvisation is treated as the epitome of individual agency (Mannucci et al., 2021). The COVID-19 crisis has shown that the capacity to improvise in the face of a disruptive challenge is also informed by structural location (including race, gender and age) and felicitous conditions (affordances of resource access, existing social networks, individual empowerment) but also exacerbating the handicaps of pre-existing vulnerabilities and inequalities, and so on.

The pandemic has turned the world upside down (Grint, 2020) – for some more than others – and offered numerous expressions of the roles of leaders and institutions in handling a major crisis. Different leaders have used their power to respond differently, from Jacinda Ardern’s politics of care and compassion (Simpson et al., 2022; Tomkins, 2020) to Narendra Modi’s escalation of Hindu nationalism (Prasad, 2020). The crisis context has been revelatory of how a leader’s caring approach affects action or the lack of it. These observations indicate the significance of power and paradox.

### Power

The pandemic provided an opportunity to highlight a hereto underexplored aspect of improvisation, power relations, where the need to improvise was seen to afford greater legitimacy to managerialism, eroding democratic deliberation, autonomy and individual freedoms. Unexpected events, even in the face of inaction, always have effects as existing frames, meanings and codes cannot process them; in the face of unanticipated and initially inscrutable uncertainties improvised responses offer no guarantee of success. At a time when organizations did not have the luxury of waiting to respond until a plan was composed, improvisations were forced through and frequently imposed by managerial decree. These mandated improvisations by fiat have invisible consequences.

Attending to these requirements makes it possible to highlight how management might legitimize improvisation in a manner that suppresses (but is unable to destroy) learning without giving rise to pragmatic paradoxes, where a managerial order must be disobeyed to be obeyed (Berti and Simpson, 2021) and learning is valid only when mandated from the top. Yet, disruption creates fissures and opens space for bottom-up approaches as well. Where there is a dearth of compassionate leadership and an excess of the certainties of fiat in face of the scarcity of resources, people will learn to improvise and take initiatives into their own hands. It is in moments when individuals or collectives are unable to cope or request support that critical events such as pandemics can give rise to heroic responders who risk their own well-being to address the suffering of others. These can manifest as bottom-up/alternative approaches to improvisation, for example, improvisation as compassion initiatives to support individual citizens or co-workers or they may be community/non-community/non-hierarchical initiatives, such as anti-racist organizing as expressed by the #BlackLivesMatter movement that gained momentum during the lockdown. However, some of these heroic responders had no choice. For example, healthcare workers were obliged to turn up to work even if they did not have adequate PPE, drawing attention to the need to examine how improvisation acts on existing vulnerabilities in the health and social care workforces and the normal assumptions of supply chains and just-in-time modes of distribution. Workers in other affected industries such as hotels and airlines saw their business suspended and were forced to cultivate improvisation as a form of resilience (Lombardi et al., 2021), while organizations and governments also improvised to keep people in work.

### Paradox

Paradox refers to persistent oppositions that define organizational dynamics (Berti et al., 2021; Schad et al., 2016; Smith and Lewis, 2011). Managing a crisis such as a pandemic requires massive improvisation, as shown by the case of Taiwan’s Central Epidemic Command Center (CECC). The Centre produced and implemented a list of 124 measures between 20 January and 24 February 2020, many of which were improvised. As the case of the CECC demonstrates, large-scale effective improvisation requires careful preparation that can diligently attend to democratic freedoms. The paradoxical interplay of planning and spontaneity in improvisation has been discussed (Clegg et al., 2002). The most effective improvisation is more than a mere spontaneous reaction to events. It refers to a category of behaviours (Weick, 1998) that includes spontaneity in conjunction with careful planning in preparation for expected crises. Of course, no crisis is entirely predictable in what to expect, which means that improvisation is always needed, which some collectives will meet with better preparedness than others. Taiwan responded better than most countries. It improvised fast because it was prepared to improvise (Wang et al., 2020). The permanent dialectic between planning and spontaneity imbues the improvisational process with paradoxical attributes (Clegg et al., 2002). Improvising is more than mere responding, as it involves a measure of preparation: one cannot improvise over nothing. The pandemic illustrated this tension, with organizations as regulated as hospitals being forced to improvise as their everyday practices and assumptions were disrupted (Lloyd-Smith, 2020; Wiedner et al., 2020).

## Breaking new ground: the contributions in this special issue

Learning-through-improvisation during a pandemic was the theme of this special issue proposal: *in the face of a major improvisation enabler, how do power circuits respond and with what consequences? How can learning occur in contexts of extremity, risk and urgency?* This major question has been approached from a variety of angles by the scholarship represented in this special issue. It includes scholarly submissions representing theoretical and empirical methodologies, with analytical foci ranging from the individual level to the group level, to organizational levels and leadership. In all, five papers are included, constituted by the scholarly efforts of 10 academics. Each paper furthers improvisation scholarship in new directions.

Meisiek and Stanway observe in ‘Power, Politics, and Improvisation: Learning during a Prolonged Crisis’ that empirical research on ‘learning from improvisation remains scarce’. They sought to address this gap through a longitudinal study in a university context that partly coincided with the COVID-19 pandemic. Their findings reveal a succession of changing organizational attitudes towards a specific communication technology, one widely used by international students but dismissed by university administrators as antithetical to its rational organizational processes. Tensions of stability and change saw institutional adoption of the technology morph from initial resistance, to being tolerated as a responsive measure in a rapidly changing environment, to being accommodated through semi-structures that allowed improvisational space. Surprisingly, in what might otherwise be viewed as a case of ‘the tail wagging the dog’, the authors observed that power and politics played a greater role in this improvisational morphing than did learning. Furthermore, learning was found to follow improvisation, rather than the other way around. The remarkable conclusion drawn is that effective improvisation drives organizational learning by challenging and disrupting entrenched power relations.

Kociatkiewicz and Kostera, writing in a refreshingly personal and poetic yet scholarly tone in ‘Longing as Learning, Learning as Longing: Insights and Improvisations in a Year of Disrupted Studies’, also explore improvisation within the context of learning within a business school. Amid the rupture caused by the COVID-19 pandemic lockdowns that made face-to-face teaching impossible, their case-study highlights paradoxical tensions of performing, learning and belonging. The pandemic produced a time when people could not be physically present at work or even walk into the street to make essential purchases. Maintaining a traditional emphasis on teaching and learning that promises career success in a competitive capitalistic environment seemed less relevant to students estranged from their peers, their instructors and their universities. Longing for connection and questioning the meaning of their education created high levels of student disengagement across the educational sector. These authors adopted teaching improvisations that facilitated learning at a more fundamental human level. By improvising with the aesthetic, they supported their students in experiencing, acknowledging and expressing their longing: a longing for human connection, for understanding. Kociatkiewicz and Kostera challenge all management educators, even in times of normality, to improvise with the aesthetic urge to infuse the student experience of management learning with a greater sense of purpose and meaning.

Lê and Pradies consider, in ‘Sailing through the storm: Improvising paradox navigation during the pandemic’, paradoxes of improvisation and power at the level of a leader’s decision-making. Employing a narrative analysis of the public utterances of a world leader they identify a politician with a paradoxical mindset whose competencies were in synergistically integrating competing but interdependent tensions in political discourse. Even so, when confronted with the tensions between societal health versus economic productivity and personal freedom imposed by COVID-19, this leader initially implemented paradox denying this *or* the other solutions, choosing one or the other of twin poles. It was only with time that the leader was able to ‘navigate’ these poles with a *both-and* paradox ‘satisficing’ orientation. Lê and Pradies’ study suggests that even when paradoxical thinking might be in a leader’s nature, when confronted with disruptive challenges in an unwieldly environment, it may still take the leader some iterations of going through the ‘fog of uncertainty’, engaging in ‘chaotic learning’ and being jolted by ‘turning points’ before an effective paradox embracing strategy can be ascertained and embraced.

Vera and Crossan similarly argue in ‘Character-Enabled Improvisation and the New Normal: A Paradox Perspective’ that a paradox mindset is not enough for an individual to ‘think through paradoxes’ imposed in a moment of crisis. Their point of departure, however, is their reasoning Central Epidemic Command Cente

that a key individual characteristic underpinning a paradox mindset is strength of character, while, at the same time, strength of character also requires the balance of apparent contradictory dimensions such as humility and drive, courage and temperance, and accountability and humanity.

Improvisation scholars have long recognized, for example, in studying the improvisation of jazz musicians or firefighters, that effective improvisation entails a combination of preparedness and spontaneity, where a gifted performance is the result of significant practice. For Vera and Crossan, it is the habituation of virtuous patterns of behaviour, thought and motivation in times of normality, which facilitates individuals in improvising to synthesize paradoxical tensions through virtue-informed priority-setting in times of disruptive uncertainty, such as the COVID-19 pandemic. The authors thereby provide a novel theory connecting three phenomena where: (a) habits of virtuous character, (b) inform improvisational strategies adopted to manage paradox tensions in an (c) uncertain environment.

Hadjimichael and Tsoukas, in ‘Phronetic Improvisation: A Virtue Ethics Perspective’, further develop the central role of the values base of improvisation at an organizational or collective level. Thus, they reason that organizational improvisation ‘is not simply a merely functional endeavour, but one that is morally committed towards accomplishing the valued ends of respective practices’. Hadjimichael and Tsoukas apply their theorizing to analysing a case study of a diary kept by Dr Ouyang, a medical doctor in New York City as she had to make life and death decisions for patients at a time when regular norms of practice were disrupted by the uncommon conditions at the height of the COVID-19 pandemic. Drawing on character virtues (‘internal goods’) she and her colleagues upheld the valued ends of their profession through improvisation. In presenting this case, Hadjimichael and Tsoukas illustrate a wider theoretical point that the habituation of virtue makes decision-making in new and conflicting situations instinctive. It is informed by an internal compass of phronesis or ‘practical wisdom’ that also facilitates coping. It might seem that their analysis reveals the enactment of virtue through improvisation at the individual level of Dr Ouyang, but Hadjimichael and Tsoukas are careful to explain that although the account is personal, the examples they choose show how virtue is enacted in an irreducibly social way through collective action. An overview of their analysis is that it delineates phronetic improvisation as informing perception of action potential arising from the ordering of priorities to balance three dimensions: (a) internal goods (valued ends), (b) institutional demands and (c) situational conditions.

## Intersectionality in divergent approaches

As indicated in Table 1 there are layers of intersectionality and divergence between these papers in terms of level of analysis, context, paradoxical tension and overall contribution to improvisation theory. Hadjimichael and Tsoukas have in common with Meisiek and Stanway the organization as their level of analysis, while they have in common with Vera and Crossan a focus on the habituation of virtuous character in facilitating prioritization of values to navigate paradoxical tensions. Taking a different approach but almost arriving at the same place, Kociatkiewicz and Kostera also implicitly explore virtue as beauty, transcendence and humanity, not as a habituated character but as something that can be leaned-into to experience paradox as profound and wondrous. Doing this is achieved through improvising with aesthetic forms that provide a sense of meaning and facilitate coping in the face of crisis. Something surprising about this virtue focus is that it represents an unanticipated contribution to improvisation scholarship emergent from the COVID-19 pandemic. While compassion was anticipated as a potentially emergent theme and is discussed at a cursory level by Hadjimichael and Tsoukas as well as Vera and Crossan, the significant link between improvisation and broader virtue had neither been anticipated nor, until now, had it been explored.

**Table 1.** Intersectionality and divergence of author approaches to improvisation in this special issue.

Authors	Approach	Context	Analytical level	Paradoxical tensions	Power	Big idea: contribution to improvisation theory
Meisiek and Stanway	Longitudinal study, interviews	Business School in Sydney, Australia	Organizational	Stability and change	Disrupting and resisting traditional institutional communications technologies in place of those favoured by students	Power and politics play a greater role in improvisational morphing than learning. Effective improvisation disrupts entrenched power relations to drive organizational learning
Kociatkiewicz and Kostera	Case study	Business School in Warsaw, Poland	Group, classroom	Aesthetic discovering of immortality in mortality, connection in disconnection	Disrupting institutional power represented in the traditional instrumental business curriculum in favour of one imbued with aesthetic sensitivity and humanity	By improvising with the aesthetic, students are able to engage and experience their own humanity as a longing for connection, for meaning and for learning
Lê and Pradies	Case study	French President Macron	Individual leader/Society	Societal health vs economic productivity and personal freedom	Institutional power of the French Presidency constrained or facilitated by the leader’s power to navigate paradoxical tensions	Even if paradoxical thinking might be in a leader’s nature, when confronted with disruptive challenges in an unwieldly environment, it may still take the leader some iterations of going through the ‘fog of uncertainty’, engaging in ‘chaotic learning’ and being jolted by ‘turning points’ before an effective paradox acceptance strategy can be ascertained and embraced
Vera and Crossan	Theory	NA	Individual	Preparedness and spontaneity	Individual power to make informed institutional decisions	Preparedness through the habituation of virtuous patterns of behaviour, thought and motivation in times of normality, facilitates individuals in improvising to synthesize paradoxical tensions through virtue-informed priority setting in times of disruptive uncertainty
Hadjimichael and Tsoukas	Case study	New York City Dr’s COVID-19 Diary	Organizational	Preparedness and spontaneity	Organizational power to make informed institutional decisions	Phronetic improvisation facilitates perception of action potential arising from the ordering of priorities to balance three dimensions: (a) internal goods (valued ends), (b) institutional demands and (c) situational conditions

Moving on from this observation, Lê and Pradies, it might be said, have in common with all these authors a concern with developing a paradoxical mindset. The mindset in question is one that enables improvisation in navigating paradoxical tensions in a manner that is generative and transcendent, rather than distressing and paralysing of agency and action potential. That is, all authors appear to question how improvisation facilitates paradox integration or transcendence. The solutions they arrive at, however, are divergent. They range from resistance over time, as discussed by Meisiek and Stanway; the eventual prevalence of an innate paradoxical mindset after a period of experimenting by addressing one paradox pole at a time, as per the analysis of Lê and Pradies; leaning into paradox tensions by improvising with aesthetics, as described by Kociatkiewicz and Kostera as well as the habituation of virtuous character described by Vera and Crossan, that, according to Hadjimichael and Tsoukas, needs to translate into phronesis to properly prioritize and address competing needs, including those of the organization.

Collectively, across all these papers it is evident that relations of power are also apparent, suggesting that power is as inherent to improvisation as it is to paradox and learning. In the theorizing of some of these scholars, such as Meisiek and Stanway, power relations are explicit with improvisation playing a role in resistance to (and resistance by) established institutional norms. In other papers, such as those by Hadjimichael and Tsoukas or Vera and Crossan, the role of power is implicit but nonetheless evident, as in the form of individual or collective agency in making informed decisions to balance priorities. The pervasiveness of power relations and paradox across each of these papers suggests that power and paradox are too important to be overlooked or ignored by improvisation scholars. Rather they should be embraced as vital to any improvisation analysis or practice.

## Further lessons from COVID-19 calling for research attention of improvisation scholars

Despite power relations being explicitly or implicitly reflected in these works, certain such relations were made salient by the COVID-19 pandemic and discussed widely in the press. These did not appear in any of the submissions to this special issue. These included improvisations in addressing the power inequities inherent in working from home, with employees blurring the boundaries of their professional and private lives while working in home–workspaces shared with family, a pressure that was felt more acutely by those from less wealthy households sharing studio or single room apartments, low-income migrant workers or those suffering from overcrowding as well as overwhelmingly female gender-specific roles. The pandemic increased the number of hours of household work and childcare undertaken by women as schools and childcare facilities closed, while domestic violence against women increased under lockdown (Office for National Statistics, 2020). Other tensions made salient by the COVID-19 pandemic relate to the equity of certain ethnic minority populations being overrepresented in essential work and thus disproportionately exposed to higher risk while keeping essential services running, while some parts of the population were shielded by remaining at home. In part, this may have had an effect of higher levels of mortality being seen among certain minority population groups. Another power imbalance is that, notwithstanding awareness and efforts to the contrary, for the most part, the cases and scholarship represented in this special issue are drawn from Western countries: Europe, the United States/Canada and Australia. Despite this special issue, improvised navigation of these power-related tensions remains a largely underexplored learning opportunity for future improvisation scholarship.

## Conclusion

In summary, this special issue studies and questions the conditions of managerially sanctioned and non-sanctioned learning, unlearning, retention and forgetfulness associated with improvisation during the extraordinary times of the COVID-19 pandemic. The global pandemic has offered an extreme environment conducive to all sorts of experiments that we can learn from, namely, how novel disruptive circumstances interact with organizational memory in terms of learning and improvisation (Moorman and Miner, 1998a). If, as has been defended, organizations need to cultivate improvisational skills (Mannucci et al., 2021), the pandemic has offered valuable lessons on the process.

The special issue extends previous explorations of improvisation that have taken place in *Management Learning*. More specifically the special issue has sought to capture the management learning generated from improvisations enacted in the New Normal (Ahlstrom et al., 2020) instilled by the pandemic. Over a significant period during the pandemic, improvisation was not a strange occurrence but part of daily life. Improvisation became part of the quotidian and exploring this reversal of figure and ground offers opportunities for learning about learnings from fast organizational responses. The pandemic was not the first nor will it be the last; over the years what has been learnt from prior cases needs to be retained and embedded in practice otherwise the next critical event with global implications will have to reinvent what was once learnt anew. Learning is great; remembering and enacting the lessons is even more important.
